# Temporal and Spatial Variation in the Population Structure of Spanish *Fusarium circinatum* Infecting Pine Stands

**DOI:** 10.3390/jof9020159

**Published:** 2023-01-24

**Authors:** David Fariña-Flores, Mónica Berbegal, Eugenia Iturritxa, Laura Hernandez-Escribano, Olga Aguín, Rosa Raposo

**Affiliations:** 1 Departamento de Biotecnología-Biología Vegetal, Escuela Técnica Superior de Ingeniería Agronómica, Alimentaria y de Biosistemas, Universidad Politécnica de Madrid, 28040 Madrid, Spain; 2 Instituto de Ciencias Forestales (ICIFOR-INIA), CSIC, Carretera Coruña km 7.5, 28040 Madrid, Spain; 3 Instituto Agroforestal Mediterráneo, Universitat Politècnica de València, Camino de Vera s/n, 46022 Valencia, Spain; 4 NEIKER, Granja Modelo—Arkaute, Apdo. 46, 01080 Vitoria-Gasteiz, Spain; 5 Estación Fitopatolóxica Areeiro, Diputación Pontevedra, 36153 Pontevedra, Spain

**Keywords:** population genetic structure, pine pitch canker disease, vegetative compatibility groups, microsatellite markers, mating type

## Abstract

*Fusarium circinatum* is an introduced fungal pathogen extended to the northern regions of Spain that causes Pine Pitch Canker (PPC) disease. In this work, we analyzed the pathogen’s genetic diversity to study changes over time and space since the first outbreak occurred in Spain. Using six polymorphic SSR markers, 15 MLGs were identified in 66 isolates, and only three haplotypes were found with frequencies higher than one. In general, genotypic diversity was low and decreased shortly over time in the northwestern regions while maintained at País Vasco, where only one haplotype (MLG32) was detected 10 years. This population also included isolates of a single mating type (MAT-2) and VCGs identified in only two groups, while isolates from NW regions were of both mating types and VCGs represented in 11 groups. The existence of haplotype MLG32 maintained on time and widely distributed suggests its good adaptation to the environment and the host. Results showed that the pathogen in País Vasco remains clearly differentiated from other northwestern populations. This fact was supported with no evidence of migration among regions. Results are explained by the asexual reproduction, but also selfing at least to a lesser extent that leads to identification of two new haplotypes.

## 1. Introduction

The introduction of an exotic pathogen into a new area leads to the establishment of new biotic interactions, giving rise to a process of coevolution between the host and the pathogen [1,2]. For the pathogen, the ability to establish itself is closely linked to its ability to survive and reproduce within the host [3], and therefore to its pathogenicity (defined as the ability of a pathogen to cause disease in a particular host [4]) and virulence (defined as the degree of damage to the host [4]). Genetic variability in the pathogen population is essential to ensure the successful adaptation of the pathogen to the new environment [5].

*Fusarium circinatum* is an introduced pathogen currently established in Europe in Spain and Portugal infecting *Pinus radiata* and *P. pinaster* [6]. This pathogen causes the Pine Pitch Canker (PPC) disease on mature trees, *P. radiata* being one of the most susceptible species while *P. pinaster* is moderately resistant to the disease. Typical symptoms are sunken cankers on branches and stem with abundant resin, and wilt and necrosis of needles above the infection point [7]. *Fusarium circinatum* also infects seedlings in nurseries causing pre- and post-emergence damping-off [7]. PPC is one of the most important diseases worldwide [7,8]. In the European Plant Protection Organization (EPPO) territory, it is a disease included in the A2 list (includes pests locally present in the EPPO region), and in the EU, it is regulated as a quarantine pest (Commission Decision 2007/433/EC and 2019/2032/EC) in order to prevent the introduction and spread of *F. circinatum* in the EU.

DNA-based studies have been carried out on *Fusarium circinatum* populations worldwide to analyze their structure and infer the reproduction mode and pathways for pathogen introduction and migration [9]. First studies done in Japan and California showed low levels of genetic diversity and concluded the lack of sexual reproduction [10,11,12]. A global analysis including isolates from different geographical locations [13] revealed that populations from Mexico, Portugal, South Africa, Uruguay, and the USA were more diverse than those from Spain, Chile, France, and Japan. MLGs identified in the USA were spread worldwide which suggests a common source for introductions. Regarding sexual reproduction, occurrence was rejected for populations from the USA and Spain, but not from Chile and South Africa. However, in a recent study in South Africa population [14], ongoing sexual reproduction was rejected, indicating that the highly diverse genetic diversity detected was probably a consequence of multiple different introductions.

Genetic diversity analysis revealed that Spanish population is structured in two clusters, each one with a predominant haplotype (named MLG32 and MLG59), a situation compatible with two independent pathogen introductions in Spain [13]. Genetic association analysis showed linkage disequilibrium and clonality in the population, supporting the lack of sexual reproduction in the *F. circinatum* Spanish population. Both mating types, MAT-1 and MAT-2, were found, although one of the two clusters (with predominant haplotype MLG32) only contained isolates of mating type MAT-2 located in the northeastern region (País Vasco). A further study with isolates representing the main haplotypes in each of the two clusters [15] showed that variability on life history traits such as disease severity, spore germination and sporulation do not compromise the pathogen evolutionary potential. This question is particularly important for introduced pathogens, where a reduced genetic variation may exist [1] because of the small population size when established in a new environment.

There are different mechanisms potentially associated with significant changes in genetic variability of *F. circinatum*. Heterokaryons are originated through: (1) hyphal anastomosis of isolates of the same vegetative compatibility group (VCG) [16] and (2) selfing, which results from fusion of haploid cells produced by the same individual [16,17]. For *Fusarium circinatum* the sexual state has not been reported in nature [9], and VCGs have been determined in some populations. In southeastern USA, where PPC disease was first described in 1946, a total of 45 VCGs were found [11], while in California, where disease was reported 40 years later, eight VCGs were identified [18]. In northeastern Spain, VCGs were determined in isolates from País Vasco [19] revealing the existence of only two vegetative compatibility groups (VCGs) of the same mating type. No other study to determine VCGs in a broader Spanish population including other regions but País Vasco has been carried out.

Regarding selfing mechanism, it is differentiated haploid from diploid selfing [17] with different consequences on the genetic population structure [17]. Diploid selfing is the fusion of haploid cells originated from meiosis in the same individual (unlike outcrossing, where haploid cells are from different individuals). In heterotallic fungi such as *Fusarium circinatum*, haploid selfing is prevented [17] since the haploid cells are originated by mitosis and do not carry different alleles for the mating types that are required for the fusion of the cells. Furthermore, there is growing evidence that cryptic sex occurs in fungi for which sexual reproduction has not been reported [17,20,21]. In the case of *Fusarium circinatum*, the sexual stage has not been reported in the field [9], but outcrossing was successfully conducted under laboratory conditions [18,22,23], producing perithecia with viable ascospores that indicates that both mating types are functional and sexual reproduction is possible. These three types of reproduction, haploid and diploid selfing, and outcrossing, in which meiosis and recombination are involved, are considered different modes of sexual reproduction occurring in fungi [17].

Population genetics studies provide insight into the evolutionary process and changes in gene frequencies that shape pathogen population structure [24,25]. Introduction of a pathogen into a new area leads to new host-pathogen interactions usually without a coevolutionary history, which have impact on their distribution and abundance and possible changes in genotype frequencies [1,2]. Moreover, pathogens must evolve constantly to adapt to changes in their environment and survive [1,3]. *Fusarium circinatum* is extended in Spain to the Atlantic coast [26] where most *Pinus radiata* plantations are grown. It is the only exotic species of pine planted in Spain and cohabits in this area with *P. pinaster*, a native Mediterranean species. Moreover, some new environmental situations have been identified after its introduction [15], such as the differential genetic resistance that pine species have in Spain (*P. radiata* more susceptible than *P. pinaster* [27]) that exert different pressure to the pathogen, or the management measures taken to mitigate the damage.

The objective of this work is to quantify the genetic diversity of Spanish *Fusarium circinatum* populations infecting pine stands and to determine how this variation is distributed in a temporal and spatial scale. The aim is to understand the biological significance of the variation detected and to make inferences about the evolutionary processes that may influence the population structure.

## 2. Materials and Methods

### 2.1. Sampling and Fungal Isolation

**Figure 1:**
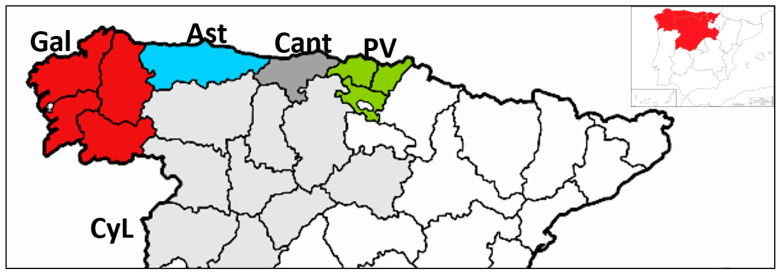
Geographic origin of the Spanish *Fusarium circinatum* isolates used in this study. Gal: Galicia; Ast: Asturias; PV: País Vasco; Cant: Cantabria; CyL: Castilla y León.

A survey was done in 2018–2021 in northern Spain. *Fusarium circinatum* isolates were obtained from cankers of forest trees showing symptoms of PPC. Small pieces of samples were cut from the margin of symptomatic tissue and plated on Fusarium Selective Medium (FSM) [28]. Isolation plates were incubated at 22–24 °C in darkness. All isolates of *F. circinatum* were identified based on morphological features and molecular methods. For morphological fungal identification, putative colonies of *F. circinatum* growing on FSM were transferred to plates with Spezieller Nahrstoffarmer Agar (SNA) medium [29] incubated for 7–10 days at 25 °C, and then, microscopically inspected for the formation of coiled sterile hyphae (circinate hyphae) characteristic of *F. circinatum* [30]. Molecular identification of *F. circinatum* was done by PCR using specific primers CIRC1A (CTTGGCTCGAGAAGGG) and CIRC4A (ACCTACCCTACACCTCTCACT), as described by Schweigkofler et al. [31] in a final volume of 25 µL with 1 µL of DNA template. PCR product was visualized in a 1% agarose gel (Agarose MS-12, Condalab, Madrid Spain) stained with RedSafe (RedSafe Nucleic Staining Acid Solution, Intron Biotechnology, Seongnam-si, Republic of Korea) under UV light. A 100 bp ladder was used as molecular weight marker (Biotools, Madrid, Spain). *F. circinatum* isolates produced a 360 bp amplicon. In total, 29 *F. circinatum* monosporic isolates were obtained, 5 isolates from Galicia, 9 from Asturias, and 31 from País Vasco (Table 1, Figure 1). A unique isolate from each tree in an affected stand was selected for the study.

To analyze genetic variation of pathogen population in a temporal scale, we used a set of monosporic isolates maintained at the Instituto Agroforestal Mediterráneo, Universitat Politècnica de València, collected from a first survey carried out in 2004–2011, soon after the first official report of the pathogen in Spain [13,32]. Isolates were selected to come from pine stands localized at the same regions as those currently collected from. In order to determine if spread of individuals from nurseries has occurred over time, a set of isolates from nurseries obtained in the first survey were also included and considered as a single population in the analysis (Table 1, Figure 1). Selection included all haplotypes identified in the first survey [13].

### 2.2. DNA Extraction

Fresh mycelia growing on PDA (Condalab, Madrid, Spain) plates at 22 °C was scraped and ground to a fine powder under liquid nitrogen in a mortar. DNA was extracted using the E.Z.N.A. Plant DNA kit (Omega Biotek, Norcross, GA, USA) or NZY Plant/Fungi gDNA Isolation kit (NZYTech, Lisbon, Portugal) following the manufacturer instructions. DNA obtained with the latter kit was used only for microsatellite analysis. DNA quantity and quality was measured using a spectrophotometer (NanoDrop 2000, ThermoFisher Scietific, Waltham, MA, USA). For molecular marker analysis, only high-quality DNA was used, with absorbance ratio 260/280 and 260/230 > 1.8.

### 2.3. Mating Type Determination

Idiomorphs MAT-1 or MAT-2 were determined for each isolate. A multiplex PCR assay was performed using primers MAT1p1 (AGAAACTGACTGATACATCAAGGGG)-MAT1p3 (TCATAAGAAGTGTTGAAGGAATCACAG) and GcHMG1 (CTTTACCGTAAGGAGCGTCACCAT)- GcHMG2 (TGATCCGCCATCTGCTTGTAGAGT) for alleles MAT-1 and MAT-2, respectively, as described by Wallace and Cover [33] and Schweigkofler et al. [31]. A final volume of 25 µL with 1 µL of DNA template per reaction was used following amplification conditions described by Berbegal et al. [13]. PCR products were visualized as explained above. MAT-1 isolates amplified a 380 bp fragment, while MAT-2 produced a 190 bp amplicon.

### 2.4. Haplotype Determination

Microsatellite genotyping was carried out by AllGenetics and Biology SL(A Coruña, Spain). Briefly, samples were genotyped using a set of seven microsatellites previously designed for *F. circinatum* [34] and selected by Berbegal et al. [13] for polymorphism, specific amplification, and reproducible allele calls. The seven primer pairs (FCM-2, FCM-4, FCM-6, FCM-7, FCM-19, FCM-25, and FCM-26), organized in three multiplexes, were tested and checked for polymorphism in 65 individuals of *F. circinatum* (Table 1). Each PCR reaction was performed in a final volume of 12.5 µL with at least three oligonucleotides: (1) a microsatellite-specific forward primer; (2) a microsatellite-specific reverse primer with an oligonucleotide tail at its 5′ end; and (3) a fluorescently labeled oligonucleotide identical to the 5′ tail of the reverse primer. The oligonucleotide tails used were the universal sequences M13 (GGA AAC AGC TAT GAC CAT), CAG (CAG TCG GGC GTC ATC), and T3 (AAT TAA CCC TCA CTA AAG GG) which were labeled with the HEX dye, the FAM dye, and the TAMRA dye, respectively. PCR products were subsequently subjected to fragment analysis on an ABI 3730xl DNA Analyzer (Applied Biosystems, MA, USA).

### 2.5. Vegetative Compatibility Tests

Vegetative compatibility groups were determined based on pairings between nitrate non-utilizing (*nit*) mutants, following [35] slightly modified. To generate *nit* mutants, isolates were cultured on Czapeck (Cz) medium (Condalab) amended with 4% potassium chlorate at 25 °C with 12-h light cycle. Fast-growing mycelia sectors developing for 4–10 days were transferred to Cz medium. Colonies that grew as thin, expansive, and with no aerial mycelia were considered as *nit* mutants. To check for their stability, mutants were grown on PDA and then transferred to Cz and Cz with 4% potassium chlorate. For phenotyping *nit* mutants (*nit1/3* or *nitM*), they were cultured on basal medium with no mineral added [35] and supplemented with 0.2 g/L hypoxanthine. Plates were incubated at 25 °C with 12-h light cycle for 4 days. Those mutants forming thin colonies and with no aerial mycelia compared to the wild type were considered *nitM*. Otherwise, they were considered as either *nit1* or *nit3*. No attempt was done to distinguish between these two types because pairing between *nit1* and *nit3* mutants develops a heterokaryotic growth very slowly [36]. Pairings between a *nit1* (or *nit3*) and a *nitM* mutants from different isolates in all possible combinations were done to determine vegetative compatibility. Mycelial plugs placed 2–3 cm apart on Cz medium were incubated at 25 °C for 4–15 days and observed periodically for dense mycelia where colonies were in contact forming the heterokaryon. Mutant pairs that formed this dense mycelium complement each other, and thus they belong to the same Vegetative compatibility group (VCG). Pairings between a *nit1* (or *nit3*) and a *nitM* mutants from the same isolate were done to check for heterokaryon self-compatibility (HIS) which has been reported in *Gibberella fujikuroi* [35].

### 2.6. Data Analysis

Data were stratified into populations defined by time of sampling (named survey 1 and 2) and subpopulations of geographical origin (Spanish regions) where they were collected (Galicia, Asturias, and País Vasco) (Figure 1). Additionally, VCGs were determined for isolates from survey 1 collected in other Spanish regions (Cantabria and Castilla y León) (Figure 1) and not sampled in this second survey.

Genetic diversity estimated by molecular markers was based on the number of alleles and allele frequencies at each SSR locus. For each isolate, a multilocus genotype (MLG) was defined by combining data for single SSR alleles. If the MLG had been identified previously by Berbegal et al. [13], the same number was assigned. Analysis was performed for the whole set of data including all isolates and the clone-corrected dataset, where only one isolate of each MLG and sampling was included. Unless otherwise stated, most of the data analyses were performed on the full dataset using the packages adegenet [37] and poppr [38] in R version 4.2.0. A genotype accumulation curve was created to assess if markers had sufficient variability to identify all MLGs in the dataset. Shannon–Wiener Index of MLG diversity (H) [39], Stoddart and Taylor’s Index of MLG diversity (G) [40], and evenness (E_5_) [41,42,43] were used to estimate MLG diversity as suggested by Arnaud-Haond et al. [44] to account for clonality in populations. To compare genotypic richness when population size differs, we used the expected number of haplotypes (eMLG) based on rarefaction curves [43]. The standardized index of association ($\bar{r_{d}}$) ranging from 0 to 1 [45] was used instead of the index of association (I_A_) [46] because the former is not so influenced by the number of loci used in the analysis. Significance test for the hypothesis of $\bar{r_{d}}$ = 0 (value when population is under random mating and alleles at different loci are not linked) is based on the distribution expected from unlinked loci using 1000 permutations. To represent relationships between haplotypes, Neighbor-joining (NJ) dendrogram and minimum panning networks were constructed based on Bruvo’s distance [47]. Bootstrap values in NJ tree were based on 1000 permutations.

Additionally, two more tests were done to evaluate occurrence of sexual reproduction. First, the null hypothesis of mating type ratios of 1:1, which are expected under random mating, was tested. The usual chi-squared test to evaluate departure from the null hypothesis is not recommended in this case because the observed frequencies for some mating types are less than five. Instead, the theoretical probability of the observed outcome was calculated based on the binomial probability distribution on the null hypothesis with p = q = 0.5. The null hypothesis is rejected when *p* < 0.05. Second, the contribution of sexual reproduction to the genetic diversity was studied. MLGsim 2.0 (https://www.rug.nl/research/gelifes/tres/software, accessed on 10 October 2022) was used to test if identical genotypes emerged independently through random mating (Psex) based on a simulation of 1000 permutations.

Genetic differentiation between subpopulations was estimated by Hedrick’s standardized index [48] (G’st), which is not influenced by levels of within genetic variation, implemented in R mmod package [49]. Analysis of molecular variance (AMOVA) was also used to estimate the hierarchical partition of variation between surveys, geographic origin within surveys, and within regions [50]. Significance for the phi statistic (a proxy for F_ST_) is based on 999 permutations. For spatial variation study, a Mantel test was performed to test for correlations between geographic distance (calculated as Euclidean distances between geographic coordinates) and Bruvo’s genetic distance. The test was run in R using the function mantel.randtest in package ade4 [51] and its significance is based on 9999 replicates. Correlation was tested among regions within each survey and between surveys. Migrants among populations were analyzed with the software Bayesass v. 3.4 [52]. Final adjustment of mixing parameters was a = 0.5 (migration rate), f = 1 (allele frequency), and m = 0.1 (inbreeding coefficient) fixed in an iterative process, which give an acceptance rate of 0.48, 0.56, and 0.65. They could not be decreased to recommended values between 20 and 60%, likely because of the small number of samples. Estimates for the number of migrates are based on Monte Carlo Markov Chain algorithm with 106 generations.

## 3. Results

### 3.1. Genetic Diversity

**Figure 2:**
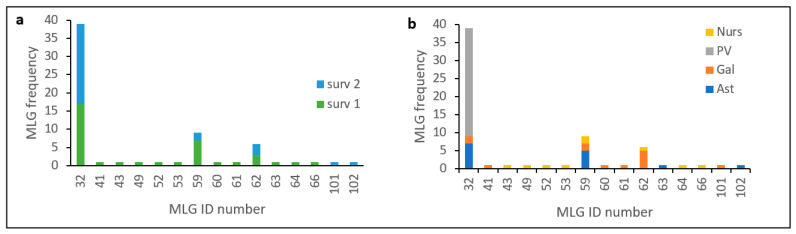
Frequency of MLG identified in the Spanish population of *Fusarium circinatum*: (**a**) by survey; (**b**) by geographic origin. MLG identification number as in Berbegal et al. [13]. MLG 101 and MLG 102 are new haplotypes detected in 2018–2021. Isolates collected from surveys (Surv) in 2004–2011 (Surv 1) and 2018–2021 (surv 2), from nurseries (Nurs), País Vasco (PV), Galicia (Gal), and Asturias (Ast). Nurseries treated as a population.

The comparison between haplotypes identified in the two surveys performed on average 10 years apart indicates that MLG32 continues to be the most frequent haplotype (Figure 2). It is represented in 39 isolates of a total 66 (59% of all isolates) collected from both surveys and distributed all over sampled regions. In this time elapsed from 2004–2011 to 2018–2021, MLG32 continues to be the only haplotype identified in the País Vasco region, and MLG59 and MLG62 are the most prevalent haplotypes identified in northwestern regions (Galicia and Asturias) (Figure 1 and Figure 2) from both surveys. We identified two new haplotypes (named MLG101 and MLG102) from samples collected in Galicia and Asturias, respectively. These haplotypes were not previously detected either in the nursery or in the field populations sampled in the first survey in 2004–2011. A total of 15 MLG were identified in 66 isolates and only three haplotypes were found with frequencies higher than one (Figure 2).

Regarding gene diversity, all loci were polymorphic except FCM-6, which did not show variation in the number of alleles (Table S1) and not included in the genetic analysis. All other loci had a number of alleles per locus in the range of 2 to 10 (Table S1), with a mean value of 3.29 alleles per locus. Two isolates displayed two microsatellite alleles per locus, one of them at three loci (FCM-2, FCM-7, and FCM-25) and the other at one locus (FCM-25) and were identified as heterokaryons. When analyzed by surveys stratified by regions, percentage of polymorphic loci was 0 (País Vasco), 86% (1_Asturias) and 71% (all others). According to the genotype accumulation curve (Figure S1), the estimated number of loci necessary to differentiate haplotypes in a random population is five indicating that our analysis with 7 loci have enough power to differentiate them. Locus FCM-25 showed the highest diversity as determined by Simpson diversity index (0.581), but this decreased most after clone correction. Locus FCM-7 was the one more evenly distributed (E_5_ = 0.961) in the whole pathogen population (Table S1). Sympson diversity index increased for loci FCM-7, FCM-19 and FCM-25 after clone correction.

Genotypic diversity in the whole population was low (H = 1.16; G = 2.03) (Table 2), as well as richness (eMLG = 3.45) and evenness (E_5_ = 0.47). Diversity and evenness decreased with time (H, G and E_5_ indices lower for the second survey compared to the first one), and richness decreased as well (eMLG = 7 and =5 in survey 1 and survey 2, respectively). When including geographic origin within surveys, the expected number of MLG in Galicia decrease from 6 to 3, while in Asturias remains at 3; H, G, and E_5_ indices (Table 2) decreased over time between regions. In contrast, only one haplotype was detected in País Vasco in both surveys (Table 2).

### 3.2. Sexual Reproduction and Clonality

Mating types (MAT-1 and MAT-2) were found in regions of Asturias and Galicia, whereas in País Vasco only MAT-2 was identified. The mode of reproduction in each geographic origin was estimated based on standardized index of association value ($\bar{r_{d}}$), calculated on the clone-corrected dataset (Table 3). The expected value of this index under null hypothesis of no linkage disequilibrium (LD) was significantly different from 0 (for Galicia in both surveys and Asturias in survey 2), rejecting random mating in these regions (Table 3). However, the observed ratios of MAT-1 to MAT2 did not confirm the absence of random mating in the *Fusarium circinatum* population. For Galicia and Asturias, ratios were 5:1 and 2:1, respectively, in isolates from survey 1, and 2:1 in those from survey 2 in both regions (Table 3). Ratios observed on the clone-corrected data were not significantly different from 1:1 ratio (Table 3) as it is expected when sexual reproduction occurs. Results from P_sex_ calculations (Table 4) are not also entirely conclusive about the lack of sexual reproduction. According to this value, probability that two individuals share either haplotypes MLG62 (for isolates from both surveys) or MLG59 (from second survey) assuming random mating is not significantly low (*p* < 0.05) and cannot be rejected. This is not concluded for MLG32.

### 3.3. Genetic Structure

**Figure 3:**
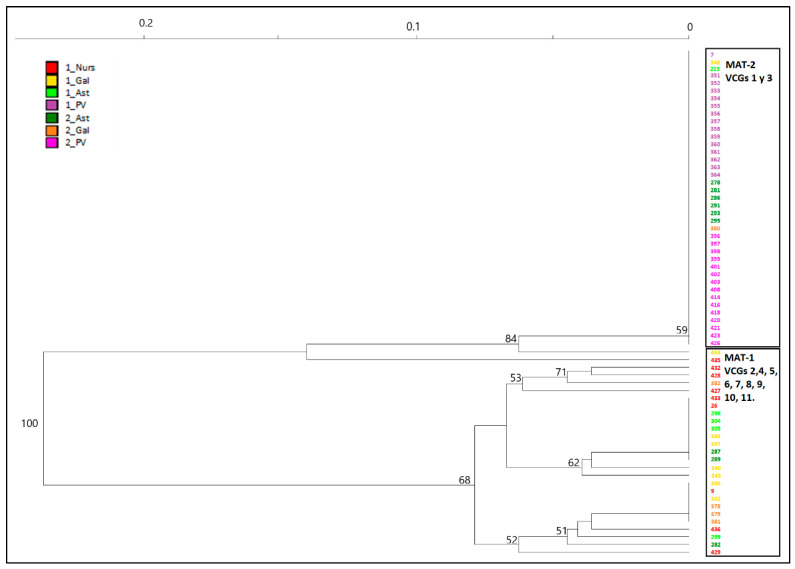
NJ dendrogram of Spanish *Fusarium circinatum* isolates based on six polymorphic SSR markers generated with Bruvo’s genetic distance [47]. VCGs (1 to 11) and Mating types (MAT-1 and MAT-2) are shown on the right side. Bootstrap values based on 1000 repetitions, shown when values > 50%. Isolates collected from surveys (1_) in 2004–2011 and (2_) in 2018–2021, from Galicia (Gal), Asturias (Ast), País Vasco (PV), and nurseries (Nurs).

**Figure 4:**
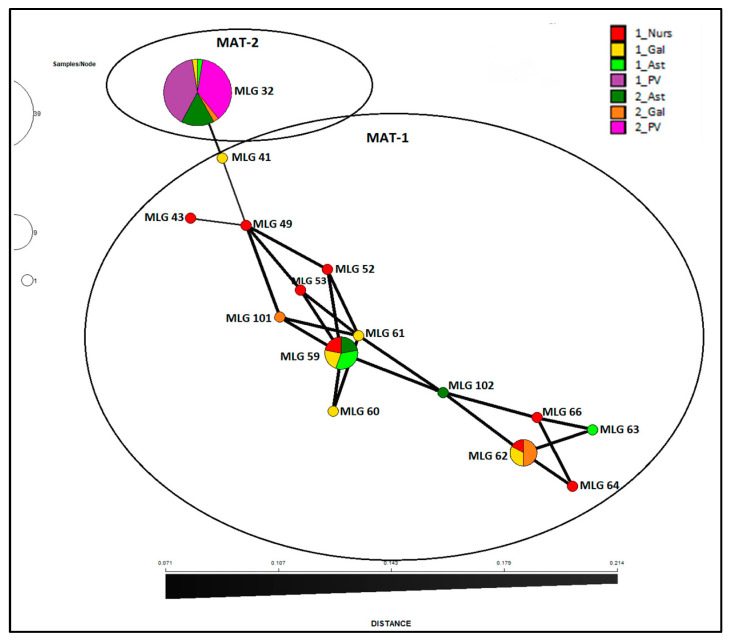
Minimum spanning network based on Bruvo’s genetic distance for haplotypes observed in the Spanish *Fusarium circinatum* population defined by survey and geographic origin. Surveys done in 2004–2011 (1_) and 2018–2021 (2_); Geographic origin: Galicia (Gal), Asturias (Ast), País Vasco (PV), and nurseries (Nurs).

No differentiation between surveys or regions within surveys were shown by AMOVA on the clone-corrected data. All variation (100%) is explained within the regions suggesting Spanish *F. circinatum* population is not differentiated (Table 5). All phi values were significantly equal to 0 (negative values are considered as 0) concluding genetic variation is not partitioned by time or regions. This pattern of variation changes when AMOVA is performed on the whole population, likely due to the weight that haplotype MLG32 has in the analysis, being the most abundant and associated mostly to only one region in both surveys. In this case, variation is significantly explained by regions nested in surveys (78%), and within regions (51%) (Table 5), but not by variation between surveys. This population structure is visualized with a NJ dendrogram with Bruvo’s genetic distance showing *F. circinatum* isolates clustered unambiguously (bootstrap value of 100%) in two major groups, one of them including all the isolates of MLG32 found in both surveys and all regions (Figure 3). Minimum spanning network based on Bruvo’s distance (Figure 4) corroborated that MLG32 was not related with the other haplotypes, being MLG41 the most closely related to it. MLG59 was the most genetically similar with the remaining haplotypes, including the new ones detected for the first time (MLG101 and MLG102).

Pairwise population comparisons of genetic diversity values (G’st index) (Table 6) indicated that population from País Vasco is differentiated from Galicia and Asturias (G’st = 0.653 and 0.592 for pairwise comparisons PV-Gal and PV-Ast, respectively in survey 1; G’st = 0.610 for both pairs in survey 2) (Table 6). Genetic diversity between northwestern regions, Galicia and Asturias, is low and decreased in survey 2 (G’st decrease from 0.138 to 0) (Table 6), likely because Asturias from survey 2 is genetically closer to País Vasco than from survey 1 as shown in the NJ dendrogram grouped by regions (Figure S2).

Mantel test showed a positive correlation between geographic and genetic distance among isolates (*p*-value = 10^−4^). A significant correlation was found between isolates from País Vasco (PV) and either Galicia and Asturias (Gal and Ast, respectively) (*p*-value = 2 × 10^−4^ and 9 × 10^−4^, respectively), but not between the populations of Asturias and Galicia in any of the two surveys (*p*-value= 0.2956 and 0.0877 for first and second surveys), corroborating that population from PV is genetically differentiated from northwestern populations (Gal and Ast). No gene flow was detected between any of the subpopulations defined by survey and origin region. According to Bayesass (Table 7), migrants were only significantly different from 0 within subpopulations.

### 3.4. VCGs

A total of 11 VCGs were identified from 106 isolates of the Spanish *Fusarium circinatum* population. Three VCGs (VCG 1, 2, and 3) were the most abundant (Table 8). The first and second largest groups were VCG 1 and 3 and included isolates from both surveys distributed over all sampled regions. VCG 2 was more abundant in isolates from first survey and distributed only on northwestern regions. Five VCGs (4, 5, 9, 10, and 11) were represented by only one isolate (Table 8). All isolates of VCGs 1 and 3 were identified with haplotype MLG32, and they were MAT-2 type. All these isolates were grouped in one of the two clusters in which population is structured (Table 8, Figure 3). Heterokaryon self-compatibility (HIS) was not detected in the Spanish isolates of *F. circinatum*, as pairings between *nit* mutants from the same isolate complement each other.

## 4. Discussion

In this work, we analyzed the genetic variation of Spanish *Fusarium circinatum* population sampled during 2018–2021 years to study changes over time since first outbreak as an exotic pathogen. Results indicate that temporal variation in genetic variability is low and decreased since 2004–2011 (Table 3). At that time, a survey was done as consequence of a severe outbreak of PPC disease in Northern regions using microsatellite (SSR) markers. Microsatellite have been the markers of choice for many population genetic studies due to their characteristics and technical advantages compared with others [53]. A faster rate of evolution makes them more sensitive to identify recent phylogeographic and demographic events, although some caution is needed when making inferences from the results because of their hypervariability [24,53,54]. A few other studies have been done but using other molecular markers [9]. Specifically, they were RFLPs [12], sequence-characterized markers used on the South African population [55] and AFLP markers used on population from País Vasco [19]. When genetic variability in the Spanish population was analyzed by regions between surveys, results indicated that variability decreases in northwestern regions while in País Vasco it is maintained (Figure 1; Table 3). This situation with differences in variability associated with geographical locations has also been described in South African populations [14]. Using SSR markers, they detected a highly diverse population in nurseries and in plantations located in some regions.

Results presented here show that Spanish population of *Fusarium circinatum* is structured in two broad groups as visualized by the minimum spanning network analysis of MLG by surveys and regions (Figure 4) as well as the NJ dendrogram including all the isolates (Figure 3). This structure was already described by Berbegal et al. [13] in an analysis that included a greater number of isolates and samples from other Spanish affected regions (Cantabria and Castilla y León), and evidenced the occurrence of at least two introductions of the pathogen in Spain. One of these clusters includes all isolates of haplotype MLG32, and MAT-2 distributed mainly in the País Vasco but also found in the Northwestern regions of Galicia and Asturias. Frequency distribution of this haplotype (Figure 2) and minimum spanning network analysis (Figure 4) shows that MLG32 presence has increased in the NW regions over time between the two surveys done (10 years apart on average), and continues to be the only one present in País Vasco.

This situation confirms that MLG32 is a well-adapted haplotype as it was shown in previous studies [15] where some pathogenic life history traits were compared among isolates. Comparison demonstrated that one isolate from País Vasco representative of haplotype MLG32 was the most virulent of those studied. This isolate caused more severe disease on *Pinus radiata* at 25 and 30 °C compared with the second most common haplotype (MLG59). Spore germination was also higher for the most common haplotype, and produced more spores at 20 and 25 °C.

Exclusive presence of MLG32 in País Vasco during these 10 years between surveys indicates *Fusarium circinatum* is inbreeding or goes under asexual reproduction, at least in this region. Only two VCGs were found in País Vasco, which contributes to a limited exchange of genetic information and maintenance of individual identity [56]. Temporal increase of VCG 1 (from 60 to 97%) respect to VCG 3 (Table 8) suggests that haplotype MLG32 and particularly VCG 1 has more fitness that other isolates and is reproducing asexually with no recombination. Other results also suggest that País Vasco is isolated genetically and there is not gene flow with northwestern regions. Mantel test showed that this region is subdivided and differentiated from the northwestern regions with no evidence of migration among populations (Table 8). Pairwise population comparisons of genetic diversity also indicated that subpopulation from País Vasco is differentiated from Galicia and Asturias.

The presence of a well-adapted haplotype together with evidence for clonality in País Vasco supports that MLG32 may have existed for some time and that this pathogen established first in this region, a hypothesis already suggested by Berbegal et al. [13]. Symptoms of PPC disease were initially observed in a nursery from País Vasco in 1990s [57], and some of these isolates were subsequently analyzed together with isolates collected during the first survey. Results confirmed that population from País Vasco at those years was already constituted by only haplotype MLG32. Iturritxa et al. [19] also reported a genetically homogeneous population using AFLPs markers in a sampling done in 2004 in País Vasco. This haplotype was later identified in other northwestern Spanish regions.

The maintenance of the population genetics in two clusters during these 10 years between surveys supports the occurrence of clonality or inbreeding and the lack of sexual reproduction. AMOVA results on the whole population show that genetic variation is explained mostly by regions, but not by time, supporting the idea of clonal organisms. However, this structure does not appear when AMOVA is performed on the clone-corrected dataset. This discrepancy in the results is probably due to the small size of the population that results from correction and point out the importance that the clones have in the AMOVA analysis.

The second cluster in which population of *Fusarium circinatum* is structured (Figure 3) is more diverse in terms of haplotypes and VCGs and includes most of the isolates of MAT-1 from NW regions (Gal and Ast in Figure 1). Here, genetic diversity has decreased between surveys (Table 3) suggesting that population is inbreeding. In addition, other results support lack of random mating. These are the standardized index of association (r _d_) (Table 4) and P_sex_ values associated with some of the three more frequent haplotypes within regions and between surveys (Table 5). Lack of significance between migrant proportions of Asturias and Galicia also corroborates inbreeding populations in these regions.

In NW regions with a greater number of MLGs and VCGs, the occurrence and identification of heterokaryons is more likely as has been the case. Two isolates from the first survey, one from Galicia and other from a nursery in Asturias, have two microsatellite alleles per locus. These heterokaryons may have originated by anastomoses of hyphae or diploid selfing (processes not necessarily exclusive [16]). Detection of two new haplotypes in the second survey, one from Galicia and other from Asturias, also suggests that these processes of hyphal fusion and/or recombination are taking place. New introductions or movement of the pathogen with plant material is not likely occurring since 2007, when the entrance and movement of material was regulated in the EU territory to prevent pathogen spread. Lack of presence of other haplotypes in País Vasco is indirect evidence of the efficiency of those regulations.

Despite the genetic uniformity found in the population of *Fusarium circinatum* in País Vasco, evidence of genetic changes was found when more intensive samplings were carried out. In an intensive sampling done in 2016 in a *P. radiata* plantation with symptoms of PPC, one new haplotype was detected by SSR marker analysis [58]. Only 1 isolate out of the 69 obtained from cankers showed polymorphism for FCM-25 loci, showing 199 bp length instead of 204 bp.

In conclusion, results presented here about the temporal and spatial variation of population structure of Spanish *Fusarium circinatum* are explained by the asexual reproduction, but also selfing at least to a lesser extent that leads to new haplotypes. The existence of haplotype MLG32 maintained on time and widely distributed across northwestern regions suggest its well adaptation to the environment and the host. Results showed that País Vasco is a region that after many years of the first disease outbreak in Spain remains clearly differentiated from other Northwestern populations. This fact was supported with no evidence of migration among regions. The low genetic diversity observed and lack of new haplotypes in País Vasco indirectly evidences that regulation within the EU territory to prevent pathogen spread is efficient. 

## Figures and Tables

**Table 1:** *Fusarium circinatum* isolates from *Pinus* spp. used for this study.

Origin ^a^	Sample Year	Isolates (N)	Tested for		
			VCG	SSR Markers	Mating Type
Galicia	2004–2011	8	8	8	8
	2018–2021	5	5	5	5
Asturias	2004–2011	5	4	5	5
	2018–2021	9	9	9	9
País Vasco	2004–2011	15	15	15	15
	2018–2021	31	31	15	31
Nursery	2004–2011	9	9	9	9
Cantabria	2004–2011	20	20	-	20
Castilla y León	2004–2011	5	5	-	5
Total		107	106	66	107

^a^ Isolates from different geographic regions were collected from pine stands. Nursery locations in Asturias were: Castropol, Siero, Grado, Navia, and Valdés. Nursery locations in Castilla y León and Cantabria were Cembrano and Torrelavega, respectively. Nursery location in Galicia is unknown.

**Table 2:** Genotypic diversity for Spanish population of *Fusarium circinatum* within subpopulations defined in a temporal scale and by geographic origin.

	Sample Size (N)		MLG No.		eMLG No.		Diversity				Evenness (E_5_) ^e^	
							H ^c^		G ^d^			
Subpop ^a^	All ^b^	C ^b^	All	C	All	C	All	C	All	C	All	C
1_Gal	8	6	6	6	6	6	1.73	1.79	5.33	6.00	0.93	1.00
1_Ast	5	3	3	3	3	3	0.95	1.10	2.27	3.00	0.80	1.00
1_PV	15	1	1	1	1	1	0	0	1.0	1.0	nc ^f^	nc
1_Survey	28	10	7	7	7	7	1.28	1.83	2.43	5.56	0.57	0.87
2_Gal	5	3	3	3	3	3	0.95	1.10	2.27	3.0	0.80	1.00
2_Ast	9	3	3	3	3	3	0.85	1.10	1.98	3.0	0.73	1.00
2_PV	15	1	1	1	1	1	0	0	1.0	1.0	nc	nc
2_Survey	29	7	5	5	5	5	0.86	1.48	1.69	3.77	0.50	0.82
Overall	57	17	9	9	3.45	6.32	1.16	1.93	2.03	5.25	0.47	0.73

^a^ Surveys 1 and 2 done in 2004–2011 and 2018–2021, respectively; Gal: Galicia; Ast: Asturias; PV: País Vasco. ^b^ Parameters calculated on the total population (All) and the clone-corrected dataset (C). ^c^ H: Shannon–Wiener Index of MLG diversity [39]. ^d^ G: Stoddart and Taylor’s Index of MLG diversity [40]. ^e^ E_5_: Evenness [41,42,43]. ^f^ nc = cannot be calculated.

**Table 3:** Standardized index of association (r _d_) and mating type in Spanish subpopulations of *Fusarium circinatum* defined in a temporal scale and by geographic origin, calculated on the clone-corrected (C) dataset.

	Mating Type Ratio ^a^		(r _d_)	
Subpop.	C	P ^b^	C	*p*-Value ^c^
Survey 1				
-Galicia	5:1	0.094	0.439	**0.002**
-Asturias	2:1	0.375	0.4	0.145
-País Vasco	0:1	0.5	-	-
Survey 2				
-Galicia	2:1	0.375	1	**0.032**
-Asturias	2:1	0.375	1	**0.035**
-País Vasco	0:1	0.5	-	-

^a^ Ratio of mating types MAT-1 to MAT-2). ^b^ Expected probability of the observed outcome based on the binomial distribution with *p* = q = 0.5 (null hypothesis of equal ratio). ^c^ Tests of significance based on 1000 permutations for null hypothesis of not linkage among loci (r _d_ = 0).

**Table 4:** Probability that a given MLG is found in a randomly mating population when observed more than once in the clone-corrected dataset of Spanish *Fusarium circinatum* population defined in a temporal scale and by geographic origin.

Subpop.	MLG ID	N ^b^	Psex	*p*-Value ^c^
1_Gal ^a^	59	2	0.09310	0.70819
	62	2	0.02659	0.37201
1_Ast	59	3	0.05078	**0.00957**
1_PV	32	15	1	
SURVEY 1	32	17	<0.001	**<0.001**
	59	7	0.00284	**0.02615**
	62	3	0.56857	0.79044
2_Gal	62	3	0.00258	**0.04528**
2_Ast	32	6	<0.0014.03 × 10 ^−13^	**<0.001**
	59	2	0.11463	0.67722
2_PV	32	15	1	
SURVEY 2	32	22	<0.001	**<0.001**
	59	2	0.69915	0.78199
	62	3	0.42808	0.60250

^a^ Surveys 1 and 2 done in 2004–2011 and 2018–2021, respectively; Gal: Galicia; Ast: Asturias; PV: País Vasco. ^b^ number of MLGs in the whole population. ^c^ *p*-value for the probability that a given MLG is found in a randomly mating population.

**Table 5:** Analysis of molecular variation (AMOVA) for Spanish population of *Fusarium circinatum* nested by surveys and geographic origin of isolates within surveys.

Source	df		Sum of Squares		Variation (%)		Phi		*p*-Value ^b^	
	All ^a^	C ^a^	All	C	All	C	All	C	All	C
Between surveys	1	1	2.262	0.544	−29.39	−4.59	0.49	−0.24	0.789	0.606
-among regions within surveys	4	4	65.189	8.79	78.43	−19.15	0.61	−0.18	**0.001**	0.902
-within regions	51	11	58.444	38.667	50.97	123.74	−0.294	−0.046	**0.001**	0.841
TOTAL	56	16	125.90	48	100	100				

^a^ Results are for the whole (All) and clone-corrected (C) population. ^b^ *p*-value is the significance for phi statistic and is based on 999 permutations.

**Table 6:** Pairwise standarized Hendrick’s index (G’_st_) of genetic diversity (2005) in *Fusarium circinatum* (clone-corrected) between Spanish populations defined in a temporal scale and by geographic origin.

Subpop ^a^	1_Gal	1_Ast	1_PV	2_Gal	2_Ast
1_Gal					
1_Ast	0.138				
1_PV	0.653	0.592			
2_Gal	−0.121 ^b^	0.198	0.610		
2_Ast	−0.159	−0.198	0.610	−0.172	
2_PV	0.653	0.592	-	0.610	0.610

^a^ Subpopulations defined by (1): 1_ survey in 2004–2011 and 2_ in 2018–2021; (2) from Galicia (Gal), Asturias (Ast) and País Vasco (PV). ^b^ Negative values are considered as 0.

**Table 7:** Migration rate means (and 95% confidence interval) within and among subpopulations^a^ of Spanish *Fusarium circinatum* defined in a temporal scale and by geographic origin, as estimated using Bayesass software.

	1_Ast ^a^	1_Gal	1_PV	2_Ast	2_Gal	2_PV
1_Ast	0.7311	0.1037	0.0334	0.0446	0.0538	0.0334
	**(0.61, 0.85)**	(−0.03, 0.24)	(−0.03, 0.09)	(−0.04, 0.13)	(−0.05, 0.15)	(−0.03, 0.09)
1_Gal	0.0435	0.7697	0.0541	0.0351	0.0442	0.0533
	(−0.05, 0.14)	**(0.65, 0.89)**	(−0.02, 0.13)	(−0.04, 0.11)	(−0.05, 0.14)	(−0.02, 0.13)
1_PV	0.016	0.016	0.8366	0.0162	0.0158	0.0993
	(−0.01, 0.05)	(−0.01, 0.05)	**(0.68, 0.99)**	(−0.01, 0.05)	(−0.01, 0.05)	(−0.06, 0.25)
2_Ast	0.0335	0.0582	0.0904	0.6996	0.0321	0.0862
	(−0.03, 0.10)	(−0.02, 0.14)	(−0.02, 0.20)	**(0.64, 0.76)**	(−0.03, 0.09)	(−0.03, 0.20)
2_Gal	0.0461	0.0841	0.0454	0.0391	0.7403	0.045
	(−0.05, 0.14)	(−0.05, 0.21)	(−0.03, 0.12)	(−0.04, 0.12)	**(0.62, 0.86)**	(−0.03, 0.12)
2_PV	0.016	0.0159	0.1072	0.016	0.016	0.8288
	(−0.01, 0.05)	(−0.01, 0.05)	(−0.05, 0.27)	(−0.02, 0.05)	(−0.01, 0.05)	**(0.67, 0.99)**

^a^ Subpopulations defined by (1): 1_ survey in 2004–2011 and 2_ in 2018–2021; (2) from Galicia (Gal), Asturias (Ast) and País Vasco (PV).

**Table 8:** Vegetative compatibility group and mating type in the Spanish population of *Fusarium circinatum* defined in a temporal scale and by geographic origin.

	VCG ^b^	Number of Isolates						% Isol ^c^	No. Reg ^d^	Mt Type ^e^	Cluster ^f^
		Gal ^a^	Ast	PV	Cant	CyL	Nurs				
Survey 1 ^a^	1	1	0	9	18	2	0	49.2	4	2	1
	2	4	2	0	0	3	1	16.4	4	1	2
	3	0	0	6	2	0	0	13.1	2	2	1
	4	0	1	0	0	0	0	1.6	1	1	2
	5	0	0	0	0	0	2	3.3	1	1	2
	6	0	0	0	0	0	1	1.6	1	1	2
	7	2	0	0	0	0	4	9.8	2	1	2
	8	1	0	0	0	0	0	1.6	1	1	2
	9	0	0	0	0	0	0	0.0	0		
	10	0	0	0	0	0	1	1.6	1	1	2
	11	0	1	0	0	0	0	1.6	1	1	2
	Total	8	4	15	20	5	9	100			
	No. VCG	4	3	2	2	2	5				
Survey 2	1	0	2	30	0	0	0	71.1	2	1	2
	2	1	0	0	0	0	0	2.2	1	1	2
	3	1	4	1	0	0	0	13.3	3	1	2
	4	0	0	0	0	0	0	0.0	0		
	5	0	1	0	0	0	0	2.2	1	1	2
	6	0	1	0	0	0	0	2.2	1	1	2
	7	0	1	0	0	0	0	2.2	1	1	2
	8	2	0	0	0	0	0	4.4	1	1	2
	9	1	0	0	0	0	0	2.2	1	1	2
	Total	5	9	31	0	0	0	100			
	Nº VCG	4	5	2	0	0	0				

^a^ Surveys done in 2004–2011 (survey 1) and 2018–2021 (2); Geographic origin: Galicia (Gal), Asturias (Ast), País Vasco (PV), Cantabria (Cant), Castilla y León (CyL), and Nursery (Nurs). ^b^ Identification number (1–11) assigned to the VCGs. ^c^ percentage of isolates of a VCG identified in that survey. ^d^ Number of regions in which a VCG was found. ^e^ Mating types 1 (MAT-1) and 2 (MAT-2). ^f^ cluster in which a VCG is grouped according to the NJ dendrogram with a 100% of bootstrap value (shown in Figure 3).

## Data Availability

Not applicable.

## Supplementary Materials

The following supporting information can be downloaded at: https://www.mdpi.com/article/10.3390/jof9020159/s1, Figure S1: Genotype accumulation curve: number of multilocus genotypes (MLG) identified based on the number of loci sampled; Figure S2: NJ dendrogram based on Bruvo’s distance of Spanish *Fusarium circinatum* populations defined by survey and geographic origin; Table S1: Gene diversity of seven microsatellite (SSR) locus in Spanish *Fusarium circinatum* population.
