# Supplementary material for: Temporal and Spatial Variation in the Population Structure of Spanish Fusarium circinatum Infecting Pine Stands
Source: J Fungi (Basel). 2023 Jan 24;9(2):159. doi: 10.3390/jof9020159 (PMC9962447; doi:10.3390/jof9020159)
Supplement: Supplementary file 1 [file jof-09-00159-s001.zip › jof-2139635-supplementary.pdf]

Supplementary Materials

Table S1. Gene diversity of seven microsatellite (SSR) locus in Spanish *Fusarium circinatum* population.

Locus	allele number (N_a)	Simpson diversity (1-D)[a]	Evenness E_5[b]
FCM-2	4	0.521	0.807
FCM-4	2	0.030	0.378
FCM-6	1	.	.
FCM-7	2	0.480	0.961
FCM-19	2	0.473	0.949
FCM-25	10	0.581	0.523
FCM-26	2	0.473	0.949
mean	3.3	0.371	0.761

[a] Simpson diversity index (1-D) [54]

[b] Evenness index [42]–[44].

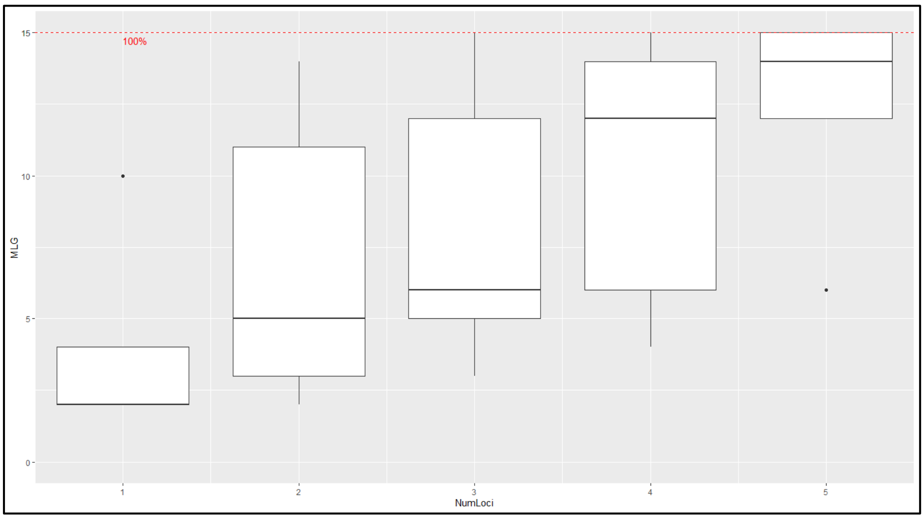

Figure S1. Genotype accumulation curve: number of multilocus genotypes (MLG) identified based on the number of loci sampled. Distribution for each boxplot based on 1000 randomizations.

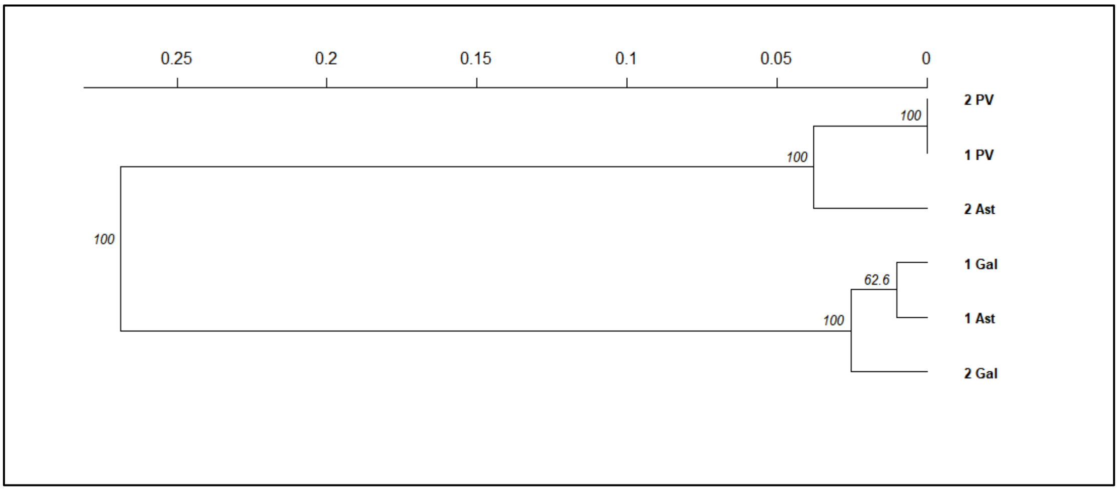

Figure S2. NJ dendrogram based on Bruvo's distance of Spanish *Fusarium circinatum* populations defined by survey and geographic origin. Surveys done in 2004-2011 (1_) and 2018-2021 (2_); Geographic origin: Galicia (Gal), Asturias (Ast), País Vasco (PV).
